# Left ventricular reverse remodeling after transcatheter aortic valve implantation: a cardiovascular magnetic resonance study

**DOI:** 10.1186/1532-429X-15-39

**Published:** 2013-05-21

**Authors:** Alessio La Manna, Alessandra Sanfilippo, Davide Capodanno, Antonella Salemi, Alessandra Cadoni, Irene Cascone, Gesualdo Polizzi, Michele Figuera, Rosetta Pittalà, Carmelo Privitera, Corrado Tamburino

**Affiliations:** 1 Division of Cardiology, Ferrarotto Hospital, (via Citelli), Catania 95100, Italy; 2 Excellence Through Newest Advances (ETNA) Foundation, Catania, Italy; 3 Radiology Unit, Vittorio Emanuele Hospital, (via Plebiscito), Catania 95122, Italy

**Keywords:** Ventricular remodeling, Transcatheter aortic valve implantation, Cardiovascular magnetic resonance

## Abstract

**Background:**

In patients with severe aortic stenosis, left ventricular hypertrophy is associated with increased myocardial stiffness and dysfunction linked to cardiac morbidity and mortality. We aimed at systematically investigating the degree of left ventricular mass regression and changes in left ventricular function six months after transcatheter aortic valve implantation (TAVI) by cardiovascular magnetic resonance (CMR).

**Methods:**

Left ventricular mass indexed to body surface area (LVMi), end diastolic volume indexed to body surface area (LVEDVi), left ventricular ejection fraction (LVEF) and stroke volume (SV) were investigated by CMR before and six months after TAVI in patients with severe aortic stenosis and contraindications for surgical aortic valve replacement.

**Results:**

Twenty-sevent patients had paired CMR at baseline and at 6-month follow-up (N=27), with a mean age of 80.7±5.2 years. LVMi decreased from 84.5±25.2 g/m^2^ at baseline to 69.4±18.4 g/m^2^ at six months follow-up (P<0.001). LVEDVi (87.2±30.1 ml /m^2^vs 86.4±22.3 ml/m^2^; P=0.84), LVEF (61.5±14.5% vs 65.1±7.2%, P=0.08) and SV (89.2±22 ml vs 94.7±26.5 ml; P=0.25) did not change significantly.

**Conclusions:**

Based on CMR, significant left ventricular reverse remodeling occurs six months after TAVI.

## Background

In patients with severe aortic stenosis, left ventricular hypertrophy is a frequent pathophysiological adaptation to pressure overload [1]. However, the enlarged myocardial cell mass and interstitial fibrosis result in increased myocardial stiffness and dysfunction [2-5]. Aortic valve replacement reduces afterload in patients with severe aortic stenosis. In recent years, transcatheter aortic valve implantation (TAVI) has emerged as a valuable alternative to surgical aortic valve replacement in patients at high surgical risk because of age and/or comorbidities [6-10]. Numerous studies have shown excellent and sustained transvalvular hemodynamics after TAVI, together with a significant improvement in symptoms and quality of life [11-13].

Hemodynamic changes that occur after TAVI have been generally evaluated by echocardiographic methods [14,15]. Cardiovascular magnetic resonance (CMR) is more accurate and reproducible than two-dimensional echocardiography in the three-dimensional volumetric evaluation of left ventricular volumes, function and mass [16]. However, there is a lack of knowledge on the use of CMR in TAVI patients for assessing the above parameters. To fill this gap, we aimed at using CMR to investigate left ventricular reverse remodeling at six-month after TAVI.

## Methods

### Patients population

**Figure 1:**
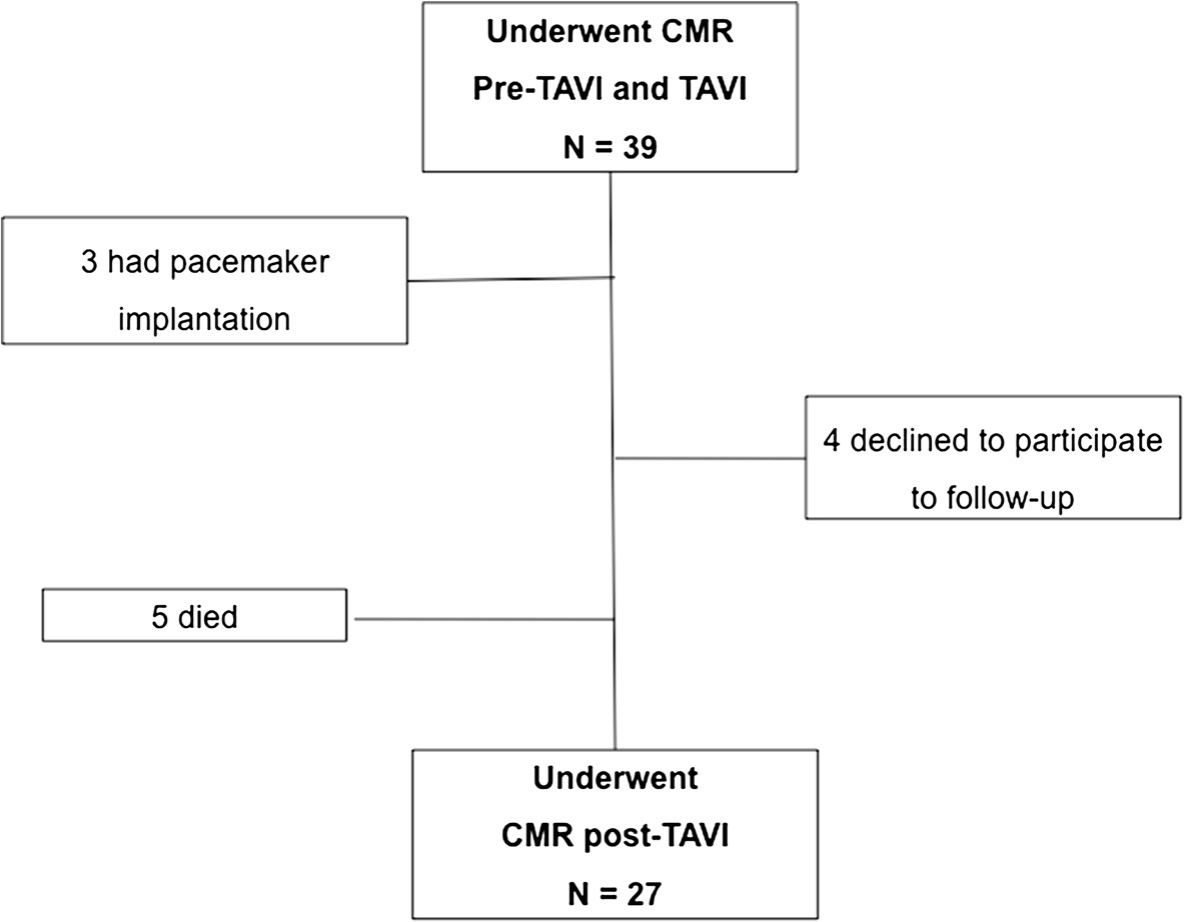
Study flow chart.

Twenty-seven patients who underwent successful TAVI using the Medtronic CoreValve (Medtronic CoreValve Percutaneous System, Medtronic CV) or Edwards SAPIEN (Edwards Lifesciences Inc, Irvine, CA, USA) bioprostheses were assessed with CMR before the procedure and at six-month follow up (Figure 1). The institutional ethics committee approved the study protocol and all patients gave informed consent. All patients were informed about the potential risks of CMR [17] and gave their written consent. Inclusion and exclusion criteria were previously reported [18].

### Study endpoints

Left ventricular mass indexed to body surface area (LVMi), left ventricular end diastolic volume indexed to body surface area (LVEDVi), stroke volume (SV), left ventricular ejection fraction (LVEF) and mass/volume ratio were assessed at baseline and six months. The occurrence of subendocardial fibrosis was assessed in terms of late gadolinium enhancement (LGE) at baseline and six months.

### Cardiovascular magnetic resonance

All CMR studies were performed with a 1.5 Tesla Magnetic Resonance Imaging scanner (Achieva, Philips Medical Systems, Netherlands) with a flexible cardiac five-element phased-array coil and a vector electrocardiogram for R wave triggering using a standard MRI imaging protocol. In brief, multiple short axis (SAX) cine images using a breath-hold steady state free precession sequence with parallel imaging (balanced Fast Field Echo (FFE); Repetition time (TR)/Echo time (TE) = 3.1/1.56 ms; slice thickness 8 mm; matrix 180 x 175 ; flip angle 60°; acquisition voxel-size = 1.78 × 1.82 × 8 mm^3^ ; reconstructed voxel-size = 1.25 × 1.25 × 8 mm^3^ acquisition; sensitivity encoding (SENSE)-factor = 2) were acquired after the acquisition of true two- and four- chamber planes for the assessment of left ventricular ejection fraction (LVEF %).The CMR images were analyzed off-line using a commercial software (Philips Medical Systems Extended MK Word Space Version 2.6.3.1) by a blinded experienced CMR reader unaware of patients’ clinical data. For assessment of left ventricular function, the end-diastolic and end-systolic cine frames were identified for each slice and the endocardial and epicardial borders were manually traced. The end-diastolic and end-systolic volumes were then calculated using the Simpson’s rule (i.e., sum of cavity sizes across all continuous slices) and indexed to body surface area. LVEF was calculated as (end-diastolic volume - end-systolic volume)/end-diastolic volume. LVMi was derived via the Simpson’s method multiplied by the specific gravity of myocardium (1.055 g/ml) and indexed to the body surface area. LVMi was divided by the LVEDVi to obtain the mass/volume ratio. Normal values used in this study for both LV systolic function and mass were those reported by Maceira et al. [19]. Left ventricular reverse remodeling was defined as a significant reduction in LVMi between baseline and 6 months.

To evaluate LGE, an intravenous bolus dose of 0.2 mmol/kg body weight of gadobutrol (Gadovist, Bayer Schering Pharma, Berlin, Germany) was administered at a rate of 3 ml/s by a power injector (MedradSpectris Solaris, Medrad, USA). Ten minutes after gadolinium injection, a ‘Look Locker’ sequence was performed to obtain the most appropriate inversion time to null the signal intensity of normal myocardium. LGE images were then acquired using the following parameters: fast gradient echo, repetition time 6.1 ms, echo time 3 ms, flip angle 25°, field of view 320 mm, slices thickness 10 mm, acquired in the left ventricular short axis over 2 RR intervals and no interslice gap. LGE was evaluated by visual assessment in short-axis slices and further characterized by spatial location, pattern, and LGE quantification (1–25%, 25–50%, 50–75%, >75%) [20].

### Statistical analysis

Quantitative data were presented as mean ± standard deviations and were compared using the Student t paired test. Univariate and multivariate correlates of left ventricular reverse remodeling were analyzed by logistic regression. To explore their significance as baseline predictors, LVMi (< or ≥ 89 g/m^2^) and LVEF (< or ≥ 55%) were dichotomyzed as previously described [19]. A two-sided p value of < 0.05 was considered to indicate statistical significance. All data were processed using the Statistical Package for Social Sciences, version 15 (SPSS, Chicago, IL, USA).

## Results

**Figure 2:**
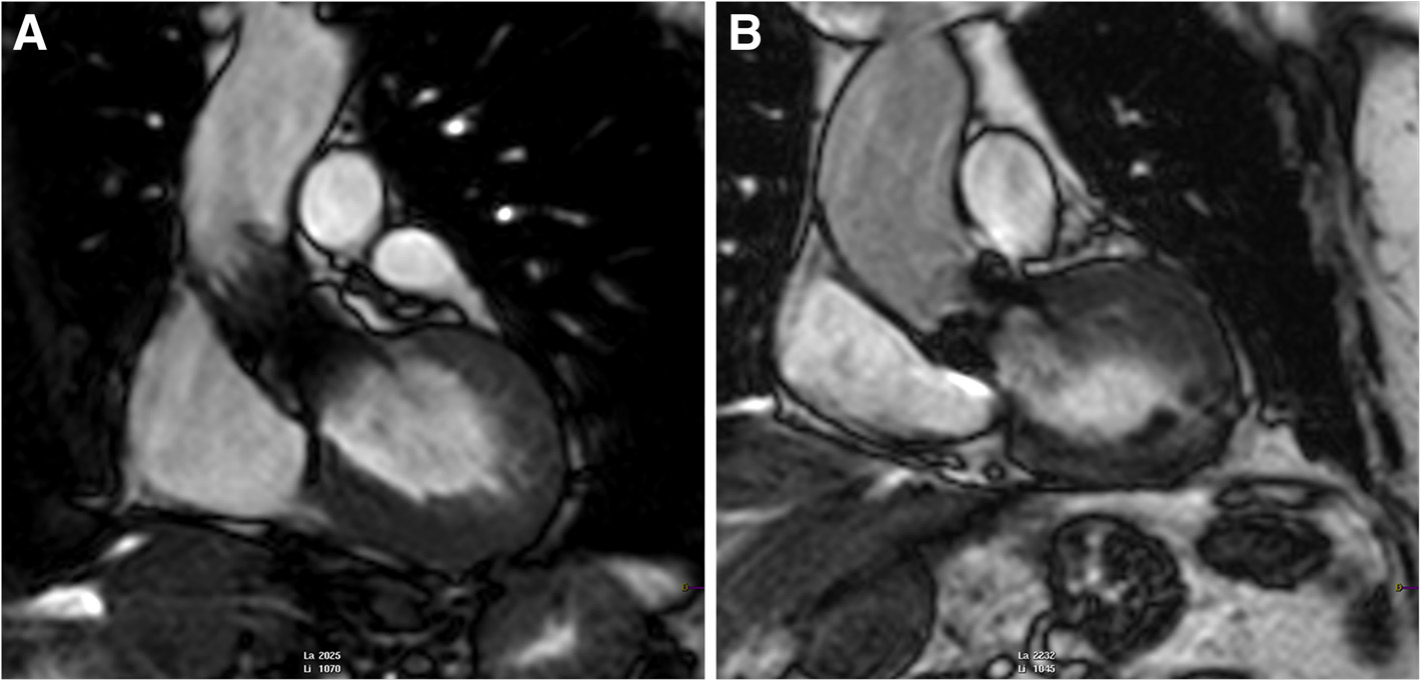
**CMR left ventricular outflow tract cine view after TAVI.** Panel **A**. Core Valve bioprosthesis; Panel **B**. Edwards SAPIEN valve bioprosthesis.

The study cohort had a mean age of 80.7±5.2 years. Detailed reasons for TAVI referral were the following: 16 (59.3%) patients had a proibitive Logistic EuroSCORE, 1 (3.7%) patient had a porcelain aorta, 3 patients (11.1%) had received thoracic radiation therapy for lung cancer, 1 (3.7%) had a severe form of diabetic lipodystrophy, 1 patient (3.7%) had cirrhosis and 5 patients (18.5%) refused surgery. All patients were in New York Heart Association class III-IV and 37% of them were previously hospitalized for congestive heart failure (Table 1). The Medtronic CoreValve and Edwards SAPIEN prostheses were used in 21 (77.8%) and 6 (22.2%) of cases, respectively (Table 1, Figure 2). All patients showed an improvement in symptoms at six-month follow-up (all were in New York Heart Association class I-II).

**Table 1:** Demographic and clinical characteristics

**Variables**	**N = 27**
Male, n (%)	10 (37)
Age, (mean ± DS)	80.7 ± 5.2
Log EuroScore, (mean ± DS)	14.9 ± 12
BMI, (mean ± DS)	27.5 ± 5.5
*Symptoms*	
Syncope, n (%)	4 (14.8)
Unstable Angina, n (%)	6 (22.3)
Hospitalization for heart failure, n (%)	10 (37)
Dyspnoea, n (%)	25 (92.6)
*RiskFactors*	
Hypertension, n (%)	23 (85.1)
Diabetes , n (%)	4 (14.8)
Hypercholesterolemia, n (%)	9 (33.3)
Smoker, n (%)	3 (11.1)
Ex smoker, n (%)	2 (7.4)
Cirrhosis, n (%)	1 (3.7)
Renal failure (creatinine > 2 mg/dL), n (%)	5 (18.5)
COPD, n (%)	7 (25.9)
Chronic obstructive arterial disease, n (%)	2 (7.4)
Previous CABG, n (%)	2 (7.4)
Previous PCI, n (%)	11 (40.7)
Untreated obstructive CAD, n (%)	2 (7.4)
Previous MI, n (%)	7 (25.9)
Previous Stroke, n (%)	3 (11.1)
Class NYHA III-IV pre-TAVI, n (%)	27 (100)
Class NYHA III-IV post-TAVI, n (%)	0 (0)
*Implanted valve*	
Medtronic CoreValve26 mm, n (%)	11 (40.7)
Medtronic CoreValve 29 mm, n (%)	10 (37.1)
Edwards SAPIEN 23 mm, n (%)	1 (3.7)
Edwards SAPIEN 26 mm, n (%)	5 (18.5)

*BMI* body mass index; *CABG* coronary artery by-pass graft; *CAD* coronary artery disease; *COPD* chronic obstructive pulmonary disease; *MI* myocardial infarction; *PCI* percutaneous coronary intervention.

### CMR outcomes

**Figure 3:**
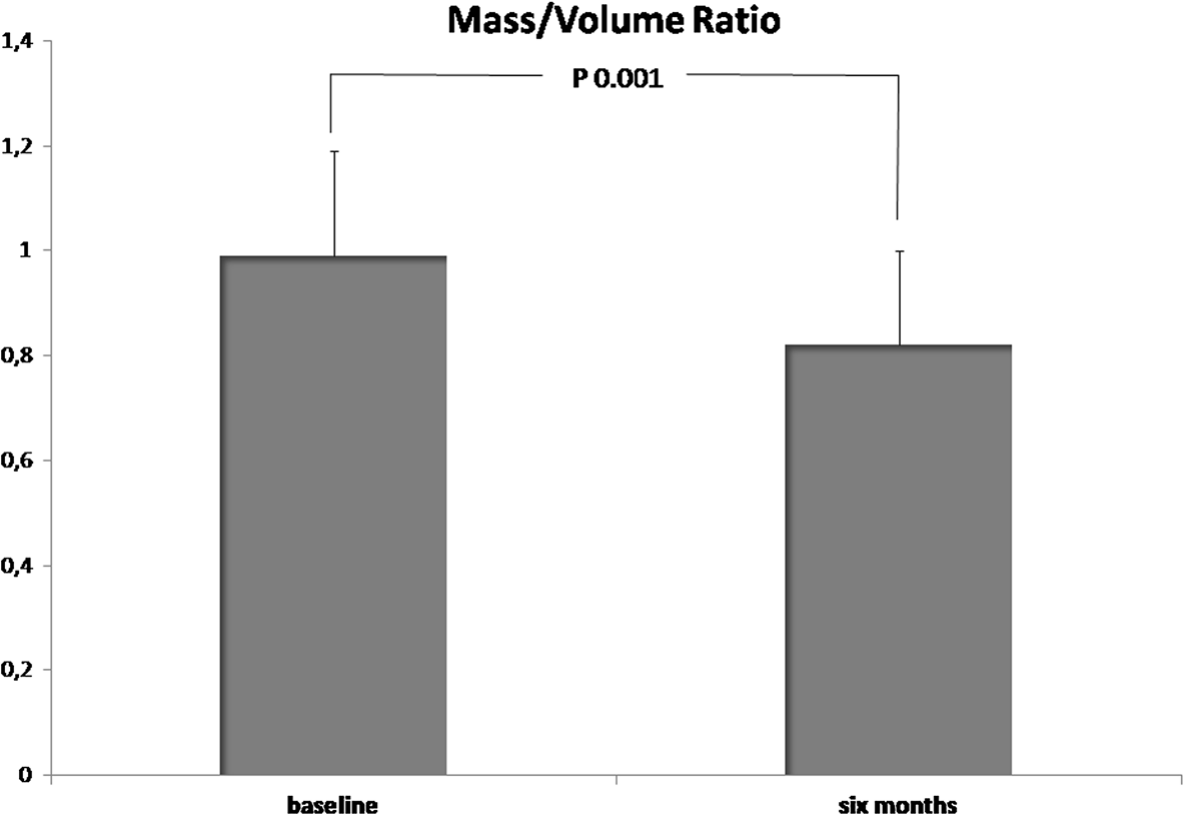
**Changes in CMR mass/volume ratio from baseline to follow-up.** The mass/volume ratio decreased from 0.99±0.2 at baseline to 0.82±0.18 at six months follow-up (P=0.001).

**Figure 4:**
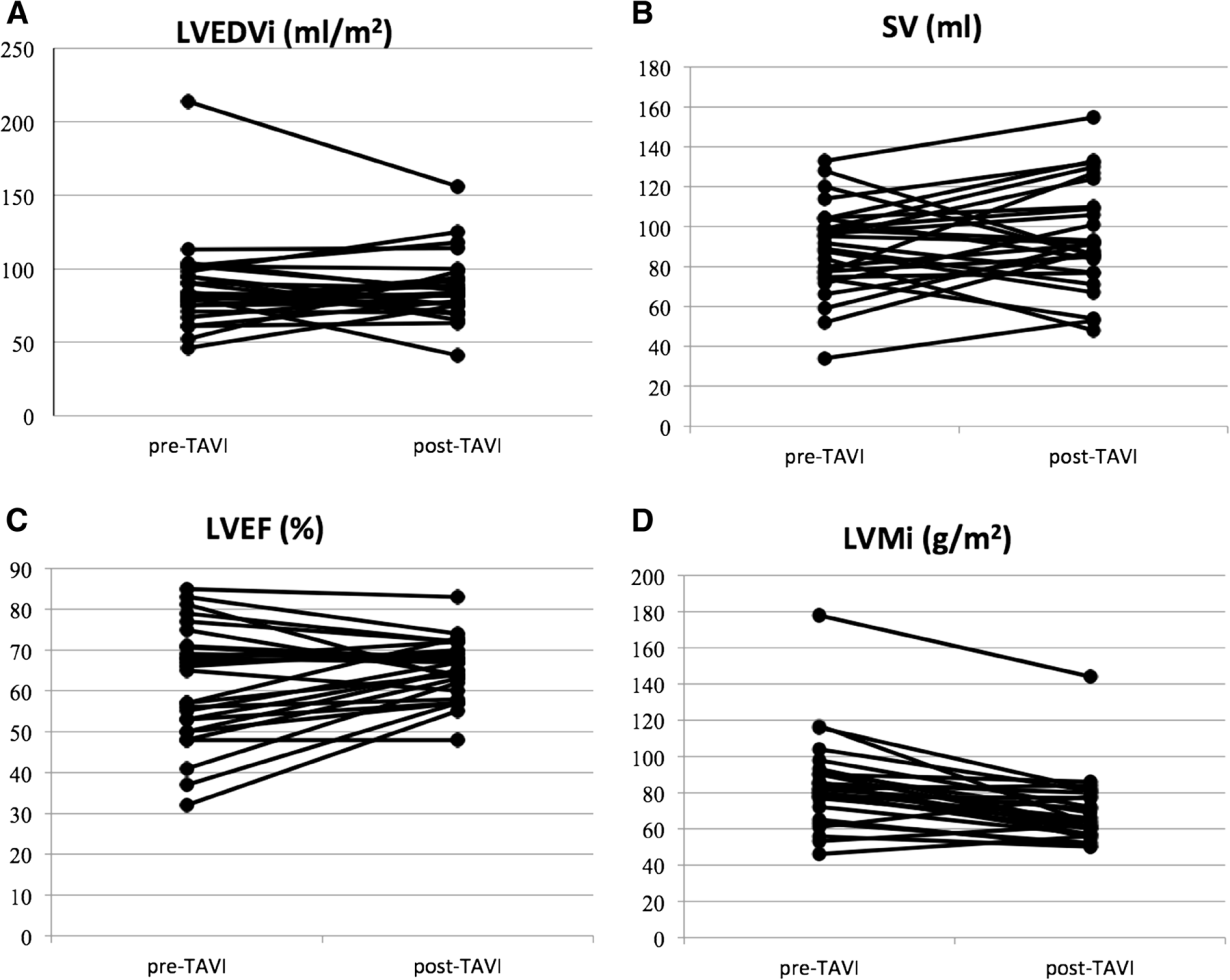
**Changes in CMR endpoints from baseline to follow up.** LVEDVi (87.2±30.1 ml /m^2^vs 86.4±22.3 ml/m^2^; p = 0.84), SV (89.2±22 ml vs 94.7 ml ±26.5; p = 0.25) and LVEF (61.5±14.5% vs 65.1±7.2%, p = 0.08), did not change significantly (Panel **A**, **B**, **C**). LVMi decreased from 84.5±25.2 g/m^2^ at baseline to 69.4±18.4 g/m^2^ at six months follow-up (p < 0.001) (Panel **D**).

All patients well tolerated CMR and no clinical adverse event was recorded during the examination both at baseline and at 6 months. The images quality was diagnostic in 100% of examinations. The mass/volume ratio decreased significantly from baseline to follow-up (P=0.001) (Table 2, Figure 3). Figure 4 displays changes in CMR endpoints from baseline to follow up. LVMi decreased from 84.5±25.2 g/m^2^ to 69.4±18.4 g/m^2^ (P<0.001). Conversely, LVEDVi, SV and LVEF did not change significantly (Table 2, Figure 4). After entering in a multivariable model all potential baseline confounding factors with p<0.20 at univariate analysis, no significant predictors of left ventricular reverse remodeling was identified.

**Table 2:** CMR characteristics in the study population

	**Baseline**	**Sixmonths**	**P**
	**N = 27**	**N = 27**	
LVEF (%)	61.5±14.5	65.1±7.2	0.08
LVEDV (ml)	151.4±50.1	151.7±38.9	0.97
LVEDVi (ml/m^2^)	87.2±30.14	86.4±22.3	0.84
LVESV (ml)	61.1±44.6	53.4±19.2	0.16
LVESVi *(*ml/m^2^*)*	35.29±24.7	30.5±11.5	0.15
LVM (g)	148.2±44.6	122.5±34.8	<0.001
LVMi *(*g/m^2^)	84.5±25.2	69.4±18.4	<0.001
Stroke volume (ml)	89.2±22	94.7±26.5	0.25
CardiacOutput (L/min)	5.9±1.4	5.9±1.3	0.98
Mass/Volume ratio	0.99±2	0.82±0.18	0.001

*CMR* cardiovascular magnetic resonance; *LVEDVi* indexed left ventricular end diastolic volume; *LVESVi* indexed left ventricular end systolic volume; *LVMi* indexed left ventricular mass; *LVEDV* left ventricular end diastolic volume; *LVEF* left ventricular ejection fraction; *LVM* left ventricular mass; *LVESV* left ventricular end systolic volume.

Because myocardial fibrosis may significantly impact clinical outcomes [21], we repeated the analysis by excluding patients with fibrosis of ischemic or non ischemic nature (N=10) and found a consistent evidence of significant LVMi reduction (83.9 ± 28.9 g/m^2^vs 69.5± 22.3 g/m^2^, P <0.001), with no significant changes for other parameters (Table 3).

**Table 3:** CMR characteristics of patients without fibrosis at baseline

	**Baseline**	**Sixmonths**	**P**
	**N= 17**	**N= 17**	
LVEF (%)	63.9±14.6	67.6±76.5	0.15
LVEDV (ml)	152.7±57.5	154.7±44.2	0.82
LVEDVi *(ml/m*^*2*^*)*	87.1±35	87.0±25.2	0.99
LVESV (ml)	60±48.8	51.1±22.7	0.27
LVESVi *(ml/m*^*2*^*)*	34.5±29	28.9±13.4	0.25
LVM (g)	148.0±50.4	124.7±41.8	0.000
LVMi *(g/m*^*2*^*)*	83.9±28.9	69.5±22.3	0.000
Stroke volume (ml)	90.9±20	98.5±28	0.57
CardiacOutput (L/min)	5.9±1.3	6.2±1.3	0.98

*CMR* cardiovascular magnetic resonance; *LVEDVi* indexed left ventricular end diastolic volume; *LVESVi* indexed left ventricular end systolic volume; *LVMi* indexed left ventricular mass; *LVEDV* left ventricular end diastolic volume; *LVEF* left ventricular ejection fraction; *LVM* left ventricular mass; *LVESV* left ventricular end systolic volume.

**Figure 5:**
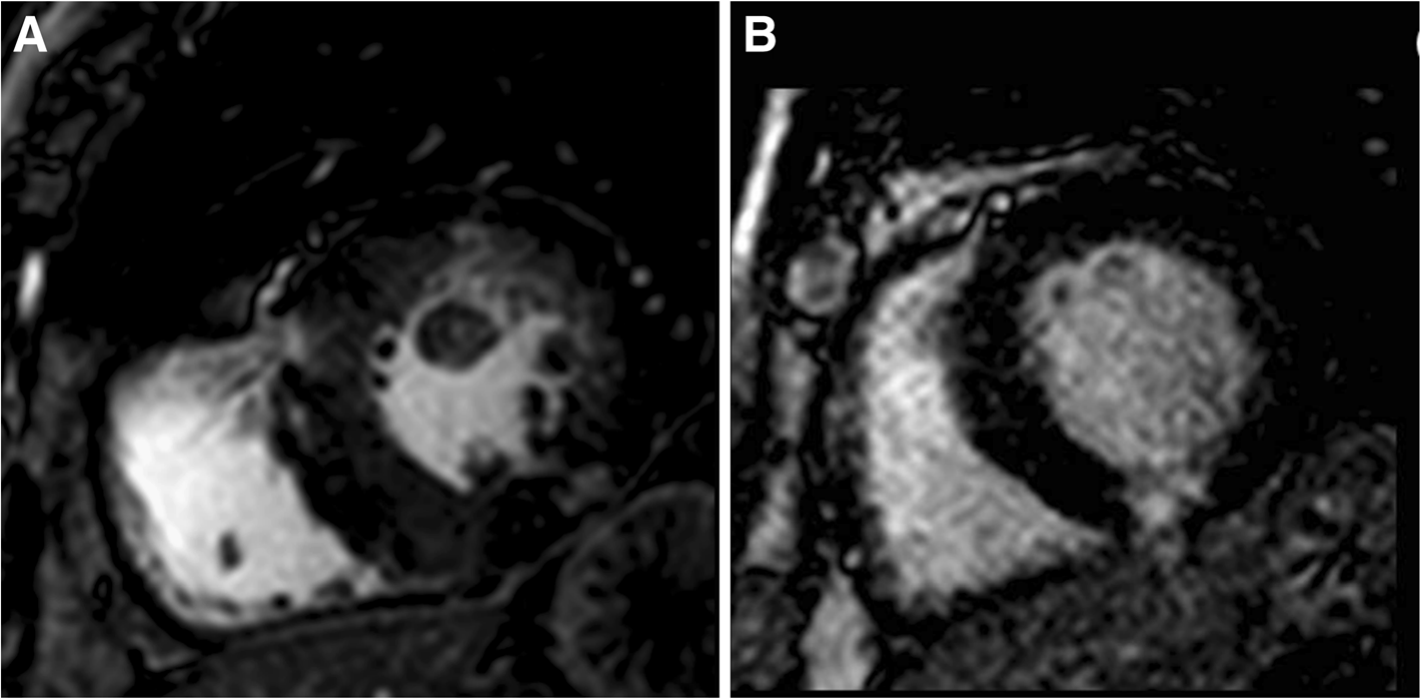
**Different patterns of LGE in TAVI patients.** Focal mid-wall fibrosis at mid segment of the inferior wall (Panel **A**). Focal transmural myocardial necrosis at basal segment of the inferior wall (Panel **B**).

Gadolinium was not administered to 3 patients due to severe renal failure (creatinine clearance <30 ml/min). At baseline, evidence of LGE was present in 10 (41.6%) of 24 cases. Causes for LGE were ischemic in 8 cases, and non-ischemic in 2 cases (Figure 5). At follow up, there were no new cases of LGE related to the intervention although one patient did have a pre-procedural myocardial infarction with new LGE at follow up.

## Discussion

Although limited by the small sample size, our study enables a deeper understanding of left ventricular reverse remodeling occurring after TAVI in a complex clinical cohort, by means of a reliable and accurate technique as CMR. In particular, at six months, we observed a statistically significant reduction of LVMi.

Regression of myocardial hypertrophy due to the decrease of ventricular afterload after surgical aortic valve replacement is a well-recognized phenomenon [5,22,23]. In particular, left ventricular mass decreases mainly within the first six months after surgical valve replacement. This observation has been also proven in studies based on CMR. In a CMR study of 24 patients, Biederman et al. [24] demonstrated that following surgical valve replacement, left ventricular mass markedly decreased at six months (157 ± 42 to 134 ± 32 g/m^2^, p < 0.005) and continued to further trend downward at 4 years (127 ± 32 g/m^2^; p =NS). Lamb et al. [25] showed that, early after surgical valve replacement, patients with aortic valve stenosis show a decrease in both LVMi, LVMi/LVEDVi ratio and improvement in diastolic filling.

Studies based on echocardiography seem to confirm that after TAVI the left ventricle undergoes a similar reverse remodeling process [26-30]. In a study by Giannini et al. comparing patients who underwent TAVI with the CoreValve bioprosthesis with those who underwent surgical aortic replacement, left ventricular reverse remodeling was found in all patients in the absence of prosthesis-patient mismatch [29]. Tzikas et al. found a significant regression in left ventricular masses in 63 consecutive patients one year after TAVI. However, regression was incomplete and was not accompanied by an improvement in left ventricular diastolic function [30]. However, it is important to underscore that transthoracic echocardiography has several limitations for the assessment of left ventricular volumes and LVEF, while CMR is currently considered the gold-standard for their assessment, especially in case of heart failure, myocardial infarction, cardiomyopathy, poor acoustic window or discrepancies between different methodologies [16]. In fact, the accuracy of left ventricular volumes and LVEF with two-dimensional echocardiography is limited by image position, geometric assumptions, and boundary tracing errors [31]. To date, few studies have been performed with CMR to assess left ventricular remodeling after TAVI [32,33]. Indeed, CMR is a noninvasive technique that allows for accurate measurement of left ventricular mass and volumes with high reproducibility without the use of geometric assumptions, thereby providing potentially more accurate information [34].

The significant reduction of the left ventricular mass observed in our study was not accompanied by a corresponding significant increase in LVEF and SV. Several studies demonstrated improved LVEF after surgical aortic valve replacement, particularly in patients with low preoperative ejection fraction [35]. However, in patients with normal preoperative LVEF, results were variable. In particular, it has been suggested that the improvement in left ventricular function is more pronounced in patients with LVEF < 50% [35]. Parameters that could influence the left ventricular reverse remodeling process include age [36], female sex [37], size of the prosthesis [38], presence of fibrosis [39] and myocardial perfusion reserve [40]. In addition, we have not observed the significant change in left ventricular volume shown in studies of the surgical aortic valve replacement [24,25,39]. This may suggest a different process of reverse remodeling between TAVI and SAVR, but the lack of a comparative control arm in our study does not allow drawing firm conclusions in this regards.

We did not collect CMR data regarding post-TAVI aortic regurgitation that could have influenced left ventricular remodeling. However, based on trans-thoracic echocardiography, no patient had a residual severe aortic insufficiency, while a mild to moderate insufficiency was present in 4 patients, a mild insufficiency in 7 and no hemodynamically significant regurgitation in the remaining population. Moreover, another major limitation of this study is the lack of information on diastolic dysfunction, flow, and changes in gradients and aortic valve areas pre and post TAVI. Finally, our study demonstrated the absence of periprocedural myocardial infarction potentially caused by the deployment of the prosthesis and possible calcium embolization.

## Conclusions

This CMR study explored the mid-term hemodynamic effects of TAVI. Our results expands on previous findings from echocardiography studies by means of a more precise and reliable imaging technique. A significant left ventricular reverse remodeling was shown at 6 months from TAVI. The implication of this finding remains unclear and should be explored in large dedicated studies with clinical endpoints.

## Abbreviations

CMR: Cardiovascular magnetic resonance; LVEDVi: Left ventricular end diastolic volume indexed to body surface area; LVMi: Left ventricular mass indexed to body surface area; LVEF: Left ventricular ejection fraction; SV: Stroke volume; TAVI: Transcatheter aortic valve implantation.

## Competing interests

Non-financial competing interests.

## Authors’ contributions

ALM conceived and designed the study, participated in data analysis, interpretation and manuscript drafting and was responsible for the final manuscript draft. AS participated in study design, data analysis and interpretation, statistical analysis and manuscript drafting. DC participated in data analysis and interpretation, statistical analysis and manuscript drafting. AS, AC, IC, GP, MF and RP performed additional data analysis. CP and CT participated in revising the manuscript critically for important intellectual content. All authors read and approved the final manuscript.
